# Nanozyme Hydrogels for Self-Augmented Sonodynamic/Photothermal Combination Therapy

**DOI:** 10.3389/fonc.2022.888855

**Published:** 2022-07-04

**Authors:** Shuntao Wang, Ning Zeng, Qi Zhang, Mingzhu Chen, Qinqin Huang

**Affiliations:** ^1^ Department of Molecular Pathology, The Second Affiliated Hospital of Zhengzhou University, Zhengzhou, China; ^2^ Department of Breast and Thyroid Surgery, Union Hospital, Tongji Medical College, Huazhong University of Science and Technology, Wuhan, China; ^3^ Department of Plastic Surgery, Tongji Hospital, Tongji Medical College, Huazhong University of Science and Technology, Wuhan, China; ^4^ Department of Radiation and Medical Oncology, Hubei Key Laboratory of Tumor Biological Behaviors, Hubei Cancer Clinical Study Center, Zhongnan Hospital of Wuhan University, Zhengzhou, China

**Keywords:** prussian blue, chlorin e6, oxygen regulation, photothermal therapy, sonodynamic therapy

## Abstract

Sonosensitizer-mediated sonodynamic therapy (SDT) has emerged as a promising anti-tumor strategy. However, this strategy of continuous oxygen consumption further exacerbates the hypoxic tumor microenvironment, which limits its therapeutic efficacy. In this study, we designed a multifunctional hydrogel (PB+Ce6@Hy) that simultaneously co-delivers nanozyme prussian blue (PB) and sonosensitizer chlorin e6 (Ce6) for the realization of photothermal therapy (PTT) and enhanced SDT. When the hydrogel reaches the tumor tissue through local injection, the 808 nm laser can induce the hydrogel to warm up and soften, thereby triggering the release of PB and Ce6. PB can interact with endogenous H_2_O_2_ *in situ* and generate sufficient oxygen to promote the Ce6-mediated SDT effect. Besides, due to the good encapsulation ability of the hydrogel, the nanomaterials can be released in a controlled manner by changing laser parameter, irradiation time, etc. The experimental results show that the PB+Ce6@Hy system we developed can generate a large amount of reactive oxygen species (ROS), which can be combined with the photothermal effect to kill tumor cells, as a result, tumor proliferation has been adequately inhibited. This combined PTT/SDT dynamic strategy provides a new perspective for Ce6-induced cancer therapy, showing great potential for clinical application.

## Introduction

Due to the limited penetration depth of light, phototherapy is not enough for deep tumors, which limits the development potential of photothermal therapy (PTT) and photodynamic therapy (PDT) (1–4). Based on this, Yumita et al. proposed sonodynamic therapy (SDT) based on PTT (5). SDT is a new treatment method for malignant tumors using a combination of sonosensitizers and low-intensity ultrasound (US) (6, 7). It has the advantages of high accuracy, deep tissue penetration, good patient compliance, and few adverse reactions. Ultrasound can penetrate deep tissue and focus on the tumor area, thereby activating the sonosensitizer, realizing ultrasonic cavitation, reactive oxygen species-induced cell damage, apoptosis, and autophagy, which provide the possibility for targeted non-invasive radical cure of solid tumors (8, 9). It is generally believed that the one of the main mechanisms of SDT is the generation of reactive oxygen species (ROS) through cavitation or cavitation-activated sonosensitizers (10). ROS can effectively destroy intracellular proteins, damage DNA, promote intracellular lipid peroxidation, further induce tumor cell apoptosis, and achieve the purpose of inhibiting tumor growth (11, 12). However, due to the malignant growth of tumor cells, solid tumor areas are usually partially hypoxic, furthermore, SDT could activate sonosensitizers agent to consume oxygen, thereby exacerbating local tissue hypoxia (13). Therefore, the tumor hypoxic state and sustained oxygen consumption during oxygen-dependent SDT severely affect the therapeutic effect.

Prussian blue nanoparticles (PB) are a class of inorganic substances assembled from transition metal ions or lanthanides through cyano bridging ligands (14). Because of its unique safety and high photothermal conversion efficiency, it has aroused great interest of researchers. PB have excellent absorption and photothermal conversion properties in the near-infrared first (NIR-I) window region (15). It is worth mentioning that compared with gold nanorods, PB nanoparticles have higher photothermal conversion efficiency, and PB nanoparticles also have better photothermal stability than general organic photothermal conversion agents (16, 17). Recently, PB has also been found to have a catalase (CAT)-like effect (18). PB can catalyze endogenous hydrogen peroxide (H_2_O_2_) in tumors to generate oxygen, which is expected to alleviate the hypoxic microenvironment of tumors. Hu et al. designed a novel PMPT nanomaterial that not only alleviates hypoxia tolerance and enhances ROS generation during photodynamic processes, but also inhibits the MTH1-regulated DNA damage repair pathway, resulting in aggravated oxidative damage and cell death (19). It has also achieved good results in animal models. Therefore, PB, which can alleviate tumor hypoxia levels, is expected to have a good synergistic effect when used in conjunction with SDT.

Although nanodrugs have greatly improved the toxicity of chemotherapeutic drugs and enhanced the enrichment of drugs in tumor sites (20), the drug accumulation in tumor tissues through nanocarriers is still less than 10% due to the complexity of the body environment (21–23). It is difficult to efficiently deliver PB nanoparticles and sonosensitizers to tumor tissues due to the interference of *in vivo* biological barriers such as liver and kidney clearance effects. Traditional drug delivery systems (i.e. intravenous injection) have a series of problems such as low efficiency of nanomaterials reaching tumor tissue, premature leakage of cargo, and profound toxicity caused by the long-term existence of the carrier in the body (24–26). Numerous studies have shown that hydrogel delivery system can prolong the sustained release time of materials, prolong the effect of materials, and improve the effect of tumor treatment (27). Adding nanomaterials into hydrogel solution by diffusion or grafting to hydrogel materials can directly hit the lesion site by implantation or injection to achieve the effect of local slow-release drugs (28, 29). As one of the popular macroscopic drug delivery carriers, light-responsive hydrogels have very broad application prospects due to their unique properties (30, 31). And the controlled release of the material can be achieved by changing the external parameters (laser power, spot size, etc.) (32). For example, Dong et al. designed a novel MXene hydrogel by combining photothermal agent MXene nanosheets with low-melting-point agarose and loading DOX into it to prepare a smart hydrogel with reversible phase transition, and *in vitro* cells experiments verified its good cell killing ability (33). These results motivate our attempts to realize enhanced SDT treatment using hydrogels.

**Scheme 1:**
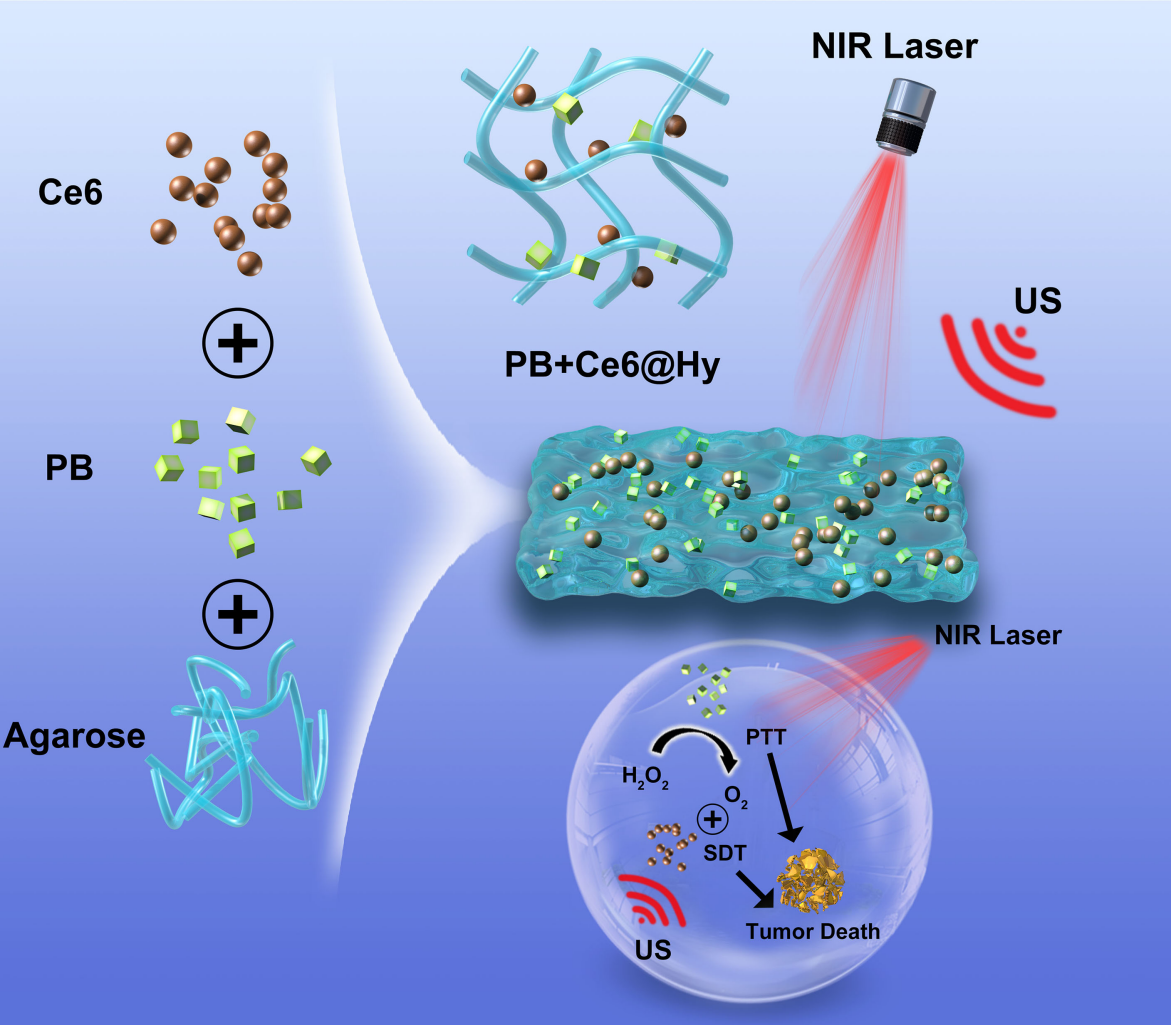
Nanozyme hydrogels for self-augmented sonodynamic/photothermal combination therapy.

In this study, we simultaneously encapsulated the sonosensitizer chlorin e6 (Ce6) and nanozyme PB into a lowmelting agarose hydrogel to prepare a multifunctional nanosystem (PB+Ce6@Hy) for enhanced SDT/PTT (**Scheme 1**). PB+Ce6@Hy could reach the tumor site by local injection, and it could be enriched here after solidification. After being irradiated by 808 nm laser, PB absorbed light energy and converts it into heat energy, causing the hydrogel to heat up and soften, then Ce6 and PB were released on demand. PB would sustainably catalyze endogenous H_2_O_2_ to generate O_2_ *in situ* to alleviate the hypoxic microenvironment, which in turn enhances the subsequent Ce6-mediated SDT. Both *in vitro* and *in vivo* experiments showed that the prepared PB+Ce6@Hy achieved the synergistic therapeutic effect of PTT and SDT on tumors. This mutually reinforcing system overcomes the deficiencies of SDT and significantly inhibits subcutaneous tumors with negligible toxic side effects. The convergence of PTT with enhanced SDT strategy provides a novel insight for Ce6-induced tumor therapy.

## Results and Discussion

**Figure 1:**
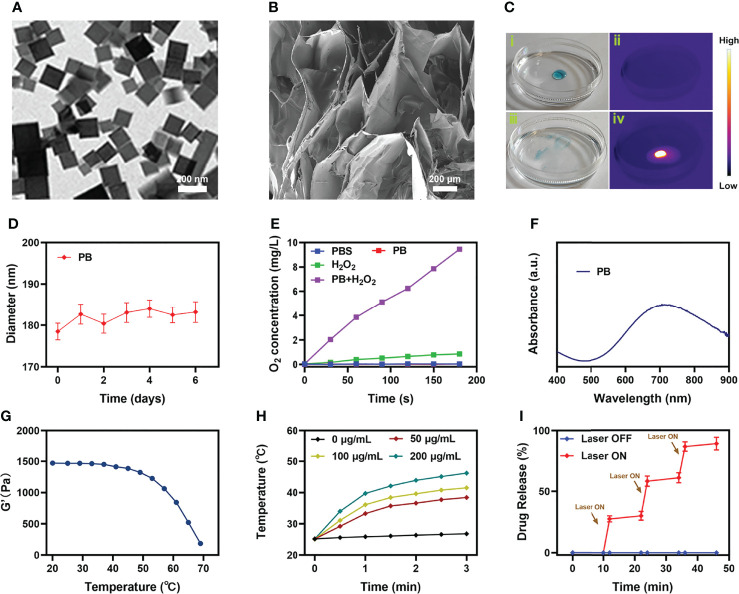
Characterization analysis of PB+Ce6@Hy. **(A)** TEM image of PB. **(B)** SEM image of agarose hydrogel. **(C)** The morphology of the prepared PB+Ce6@Hy before (i) and after (iii) 0.5 W/cm^2^ 808 nm laser irradiation for 10 min and infrared thermal images (ii, iv) of the prepared PB+Ce6@Hy following irradiation. **(D)** Hydrodynamic diameter of PB at different time points. **(E)** Oxygen generation in different conditions as measured by a dissolved oxygen meter. **(F)** UV–vis–NIR absorbance spectra of PB. **(G)** Rheological curves of agarose hydrogel. **(H)** Temperature elevation curves with the different concentration of PB at 808 nm laser irradiation. **(I)** *In vitro* Ce6 release profile in the presence and absence of 808 nm laser irradiation, with arrows being used to indicate irradiation time points.

We prepared PB according to the previous work (30), and characterized the morphology of PB by transmission electron microscopy (TEM), as shown in **Figure 1A**. Subsequently, hybrid hydrogel containing PB and Ce6 (PB+Ce6@Hy) was prepared by simple hydrothermal method. The blue PB+Ce6@Hy loses its fluidity after solidification and does not flow down with the tube wall (**Figure S1**). Scanning electron microscopy (SEM) image showed that the as-prepared hydrogels had complex three-dimensional pore morphologies (**Figure 1B**). Next, its photothermal conversion ability was verified. As shown in **Figure 1C**, the hydrogel will gradually soften and release the material after being irradiated by 808 nm laser, and the thermal energy generation ability of PB is verified by the infrared thermal image. The particle size of PB did not change significantly during the week storage period, confirming its stability (**Figure 1D**). This property is beneficial for long-term sequestration of PB in hydrogels for subsequent effects. As many unstable nanomaterials are unsuitable for long-time storage and biological applications (34). We evaluated the catalase (CAT)-like properties of PB nanozymes in aqueous solutions containing hydrogen peroxide. The results showed that PB could induce substantial oxygen production after incubation with hydrogen peroxide for five minutes (**Figure 1E**). This encourages us to use PB for enhancing SDT. UV–vis spectra of PB (**Figure 1F**) exhibited that it has strong absorbance in the NIR-I region. We prepared PB solutions with different concentration gradients to verify their photothermal properties, the control group had almost no heating effect under 808 nm laser irradiation, while the 50 μg/mL PB solution could achieve a temperature rise of nearly 15 degrees within three minutes of irradiation, and the heating effect of PB is positively correlated with its concentration (**Figure 1H**). We utilized three consecutive ON-OFF cycles of laser irradiation, that is, the PB solution was irradiated with 808 nm laser for 5 minutes and then cooled back to the initial temperature naturally, and the cycle was repeated three times. The results are shown in **Figure S2**, the heating ability of PB does not fluctuate much, confirming the photothermal stability of PB, which is also beneficial for the controllable release of PB *in vivo* for tumor therapy. Simultaneously, rheological curves of agarose hydrogel were detected, as the temperature increases (**Figure 1G**), the hydrogel will slowly transform from a solid colloidal state to a liquid state, and the storage modulus gradually decreases. We continued to study the controlled drug release kinetics of the PB+Ce6@Hy. **Figure 1I** shows the drug release curve with or without laser irradiation. When the laser switch is turned on, the temperature of the system begins to increase, and the carrier encapsulated in it is gradually released slowly. After the irradiation was stopped, the hydrogel slowly solidified and continued to encapsulate the cargo.

**Figure 2:**
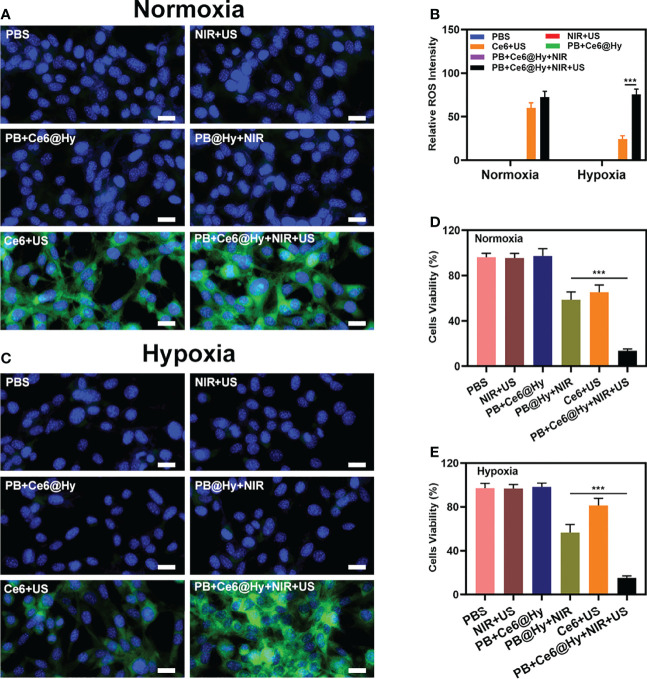
Results of *in vitro* experiments. Fluorescence microscopy images of DCFH-DA to detect intracellular ROS with various treatments under normoxic **(A)** and hypoxia **(C)** condition. Scale bar = 20 μm. **(B)** DCFH-DA fluorescence intensity after the indicated treatments. **(D)** Cell viability of 4T1 cells cultured in the presence of various formulations under normoxic condition. **(E)** Cell viability of 4T1 cells cultured in the presence of various formulations under hypoxia condition. ***P < 0.005; Student’s t-test.

Inspired by the well-characterized properties of the prepared PB+Ce6@Hy, we further explored its *in vitro* cell killing effect. SDT has a strong ability to penetrate biological tissues (35), it can concentrate acoustic energy into deeper tissues and activate the sonosensitizers (such as Ce6 in this study) in tumor tissues, ultimately playing an anti-tumor effect (36). However, SDT will continue to consume oxygen, this increased hypoxia, in turn, affects the effectiveness of SDT. So we constructed normoxic and hypoxic cell growth environments to verify the effect of PB+Ce6@Hy in regulating cell death. First, the ability of PB+Ce6@Hy combined with US and NIR to generate ROS was explored, and 2′,7′-dichlorofluorescin diacetate (DCFH-DA) was utilized as a ROS indicator. As shown in **Figures 2A, C**, the control, NIR + US, PB+Ce6@Hy and PB@Hy + NIR groups produced negligible ROS, while Ce6+US could mediate a strong green fluorescence under normoxic conditions. However, hypoxic conditions inhibited the effect of Ce6-mediated SDT to generate ROS. We quantitatively analyzed the ROS intensity under different conditions. **Figure 2B** showed that PB+Ce6@Hy + NIR + US produced bright green ROS fluorescence regardless of normoxic or hypoxic conditions, as the release of PB nanozymes upon 808 nm laser irradiation could convert endogenous H_2_O_2_ into O_2_. Since PB+Ce6@Hy has a good effect on regulating the tumor ecological environment, we continued to use the MTT experiment to test the killing effect of PB+Ce6@Hy combined with US and NIR. As shown in **Figures 2D, E**, the survival state of cells in the control group was almost unaffected. the convergence of sonosensitizers Ce6 with US in normoxic environment could achieve a moderate tumor killing effect, but the effect was adequately reduced in hypoxia. It is worth noting that PB+Ce6@Hy + NIR + US has achieved a superior therapeutic benefits and is not affected by the oxygen environment. The results of live and dead assay in the hypoxic group also fully demonstrated the killing effect of PB+Ce6@Hy system (**Figure S3**). This is attributed to the fact that PB produces O_2_ to promote SDT, and PB also has PTT effect. This dynamic oxygen-producing strategy has achieved good effects.

**Figure 3:**
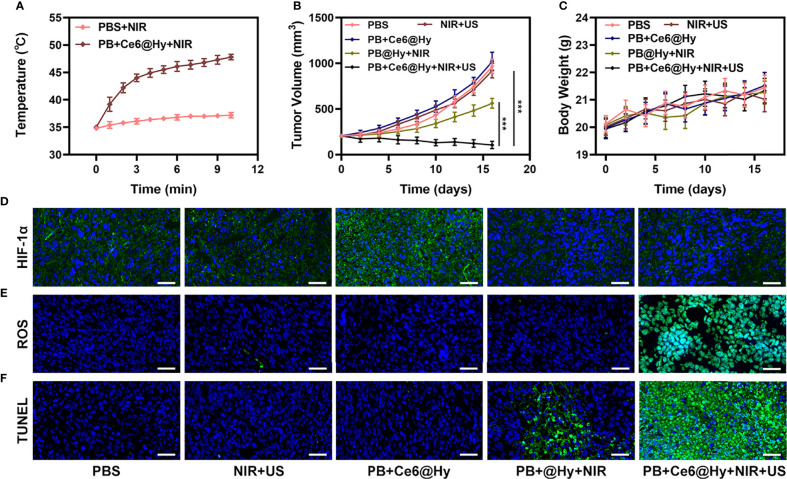
Results of *in vivo* experiments. **(A)** Temperature increases in mice implanted with 4T1 tumors following 808 nm laser irradiation (0.5 W/cm^2^) for 10 min in the indicated treatment groups. **(B)** Tumor volume change over time in groups treated as indicated. **(C)** Time-dependent body-weight curves of mice in different groups. **(D)** HIF-1α, **(E)** ROS and **(F)** TUNEL stained tumor sections from the indicated treatment groups Scale bar = 100 μm. ***P < 0.005; Student’s t-test.

**Figure 4:**
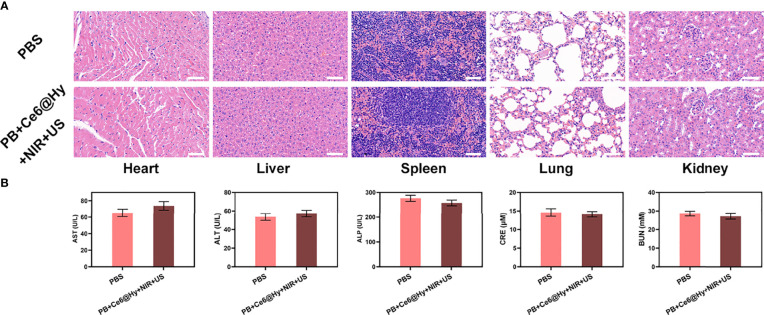
Result of *in vivo* safety experiments. **(A)** Histopathological analysis results (H&E stained images) of the major organs, heart, lung, liver, kidneys, and spleen, of mice that were exposed to different treatments 16 days post-injection. Scale bars: 100 μm. **(B)** Liver, kidney and blood function markers: AST, ALT, ALP, CRE and BUN after various treatments.

*In situ* injection of hydrogels can greatly increase the content of nanomaterials in tumor tissues. Light-responsive hydrogels are ideal biomaterials for various biomedical applications (37). The PB+Ce6@Hy we prepared has a three-dimensional cross-linked structure and good biodegradability. and biocompatibility and sensitive response to light stimuli, showing great potential in cancer therapy. We continue to explore its photothermal conversion effect *in vivo*. As shown in **Figure 3A**, PB+Ce6@Hy can achieve a good local heating effect of tumor tissue under the cooperation of 808 nm laser. PB+Ce6@Hy (10 min irradiation, 0.5W/cm^2^) could raise the tumor temperature to nearly 48 degrees. A large number of basic researches and clinical applications have proved that when tumor tissue is continuously heated for a certain period of time, the growth of tumor cells is blocked, disintegrated, and even leads to death (38). The formation of tumor thermotherapy is based on two characteristics of tumor tissue: First, there are abnormal blood vessels in tumor tissue so its heat dissipation is extremely poor (39). Local heating by appropriate methods can easily make the temperature of tumor tissue 5% to 15% higher than that of surrounding normal tissue; second, the temperature resistance of tumor tissue is significantly lower than that of normal tissue, and tumor tissue will occur at a temperature of 42°C irreversible damage (40, 41). Inspired by this result, we aimed to investigate the synergistic antitumor ability of PB+Ce6@Hy and NIR + US in 4T1 tumor mice.100 μL of 4T1 cell suspension (1×10^6^ cells per mL) were subcutaneously injected into each mouse to establish the tumor models. Subsequently, tumor growth was monitored every 2 days to assess the primary effect of the treatment system. As shown in **Figure 3B**, the injected PB+Ce6@Hy alone remained in the tumor tissue for a long time, but did not produce any tumor-toxicity, and the tumor volume growth curve was hardly inhibited. In this regard, PB@Hy containing only PB combined with laser irradiation produced a certain photothermal treatment effect. The tumor ablation effect induced by PB+Ce6@Hy plus NIR and US is the best. First, PB achieves precise hyperthermia effect, and then the softening of the hydrogel promotes the release of PB and Ce6 into the tumor site. PB can improve TME *in situ* and generate O_2_, which greatly strengthens Ce6-induced ROS production effect, tumor growth was greatly inhibited. **Figure S4** also showed that the tumor mass and volume curves are consistent. After treatment, the average tumor weight was only 0.12 g. It is worth noting that the weight of mice in each group did not increase or decrease sharply during the treatment cycle, and showed a normal growth trend, which also indicated that our treatment regimen was safe (**Figure 3C**). Hypoxia-inducible factor 1α (HIF-1α) was highly expressed under hypoxia, tumor cells under hypoxic conditions strongly expressed HIF-1α in the form of green fluorescence. We verified in an *in vivo* model that both PB@Hy and PB+Ce6@Hy systems could alleviate the hypoxic microenvironment (**Figure 3D**). In addition, PB+Ce6@Hy combined with NIR and US can also generate a large amount of ROS (**Figure 3E**) in an *in vivo* tumor model, and simultaneously increase the level of tumor cell apoptosis (**Figure 3F**). After the treatment cycle, mice in all groups were euthanized, followed by collection of major organs for further analysis and blood for biochemical analysis (**Figure 4**). The relevant results of both the experimental group and the conventional control group showed that the mice functioned normally after treatment, and our treatment method did not show short-term toxic and side effects.

## Conclusion

In conclusion, a biocompatible hydrogel containing both nanozyme PB and Ce6 sonosensitizer was constructed to achieve cooperative PTT/SDT against tumor. PB+Ce6@Hy can controllably deliver the loaded material to the tumor site and simultaneously realize on-demand release. After the hydrogel was irradiated by laser, photothermal heating was triggered, and the released PB catalyzed H_2_O_2_ to generate oxygen *in situ*, which promoted SDT. *In vitro* and *in vivo* experiments showed that PB+Ce6@Hy induced a large number of apoptosis and inhibited tumor growth without any physiological toxicity. This study provides a new mode of combination therapy and expands the application of PB nanozymes in sonodynamic therapy. Based on the fact that a single treatment method is difficult to suppress tumor cell proliferation for a long time, we will continue to develop new and safe nanozymes for multimodal treatment in the future.

## Data Availability Statement

The original contributions presented in the study are included in the article/**Supplementary Material**. Further inquiries can be directed to the corresponding author.

## Ethics Statement

The animal experiments were carried out according to the protocol approved by the Ministry of Health in People’s Republic of PR China and were approved by the Administrative Committee on Animal Research of the Wuhan University.

## Author Contributions

SW, NZ, and QZ contributed to conception and design of the study. MC and QH wrote the first draft of the manuscript. SW performed the statistical analysis. NZ organized the database. All authors contributed to manuscript revision, read, and approved the submitted version.

## Funding

This work was supported by National Natural Science Foundation of China (31800085).

## Conflict of Interest

The authors declare that the research was conducted in the absence of any commercial or financial relationships that could be construed as a potential conflict of interest.

The reviewers MS and PQ declared a shared parent affiliation with the author MC at the time of review.

## Publisher’s Note

All claims expressed in this article are solely those of the authors and do not necessarily represent those of their affiliated organizations, or those of the publisher, the editors and the reviewers. Any product that may be evaluated in this article, or claim that may be made by its manufacturer, is not guaranteed or endorsed by the publisher.
