# Supplementary material for: Nanozyme Hydrogels for Self-Augmented Sonodynamic/Photothermal Combination Therapy
Source: Front Oncol. 2022 Jul 4;12:888855. doi: 10.3389/fonc.2022.888855 (PMC9289279; doi:10.3389/fonc.2022.888855)
Supplement: Supplementary file 1 [file DataSheet_1.docx]

Experimental Procedures

**Materials and reagents.**

Chlorin e6 (Ce6), Potassium hexacyanoferrate (K_3_ [Fe (CN) _6_]) and polyvinylpyrrolidone K30 (PVP) were purchased from Shanghai Macklin Biochemical Co., Ltd. Agarose was purchased from Yare Shanghai. Reactive Oxygen Species Assay Kit, and MTT Cell Proliferation Assay Kit were obtained from Yeasen Biotech Co., Ltd (China). The other reagents used in this work were purchased from Sinopharm Chemical Reagent (China) and Aladdin-Reagent (China).

**Cell culture**

4T1 mouse breast cancer cell line was obtained from the Cell Bank of the Chinese Academy of Sciences and incubated in RPMI-1640 medium supplemented with 10% FBS in a humidified atmosphere at 37℃. Cell cultures under normoxic conditions (pO_2_: 21%) were maintained in a humidified incubator at 37℃ in 5% CO_2_ and 95% air. Hypoxic conditions (pO_2_: 2%) were produced by placing cells in a hypoxic incubator (Moriguchi, Japan) in a mixture of 2% O_2_, 5% CO_2_, and 93% N_2_.

**Preparation and characterization of prussian blue nanoparticles (PB)**

PB nanoparticles were prepared based on literature reported method[[1](#_ENREF_1)]. Typically, K_3_[Fe (CN)_6_] (226.7 mg) and PVP (3 g) were added into 40 mL ultrapure water under vigorous stirring. 35.0 μL concentrated hydrochloric acid was further added into the aqueous solution. Then, the mixture was heated at 80 °C for 20 h. After centrifugation, precipitates were collected and washed with water for several times. PB nanoparticles were obtained after vacuum freeze-drying. The size and zeta potential of PB+Ce6@Hy and PB were measured by dynamic light scattering. The morphology structures of PB was observed by the TEM (JEOL-2100). UV-vis spectra of different samples were recorded by the UV-vis spectrophotometry Lambda 35 (Perkin-Elmer).

**Photothermal ability of PB.**

Different concentration of PB PBS solution (0, 50, 100 and 200 μg/mL) were irradiated by 808 nm laser (0.5 W/cm^2^) for 3min. Through the infrared thermal imaging camera, the photothermal image of the suspension is performed at 30 s intervals.

**Preparation and characterization of PB+Ce6@Hy**

The general protocol for the hydrogel preparation is as follows. The prepared PB (10 mg/mL in PBS) and the Ce6 (10 mg/mL in DMSO) were mixed into 2% agarose solution to form PB+Ce6@Hy. Wherein the concentration of PB and Ce6 was 200 and 20 μg/mL, respectively, and the DMSO content in PB+Ce6@Hy was no more than 0.5 %.

**Rheological Test**

Rheology experiments were performed on an Anton Paar rheometer. Hydrogel samples of different temperatures were prepared and gently placed on the middle of a 15 mm diameter parallel plate with a proper gap. Dynamic oscillatory frequency sweep measurements were conducted at a 1% strain amplitude. To prevent the evaporation of water, a lid was prepared on the top.

**Evaluation the generation of oxygen**

PB were suspended in 3% H_2_O_2_ solution (8 mL), respectively. Separate PBS, H_2_O_2_ or PB PBS solutions were used as control groups. Then, liquid paraffin (2 mL) was added to reduce the influence of air. A DOG-3082 oxygen dissolving meter was used to monitor the real-time concentration of oxygen.

**Ce6 release study**

The *in vitro* Ce6 release profile from PB+Ce6@Hy was carried out. 1mL of PB+Ce6@Hy containing 20 μg Ce6 was added into culture dish. To investigate the stimuli effect of laser irradiation on the release behavior, the release experiment of Ce6 was initially performed with or without 0.5 W/cm^2^ 808 nm laser irradiation for 5 min. At appropriate time point, 100 μL of different samples were collected, and an UV−vis spectrophotometer was used to monitor the released Ce6 content.

**Intracellular reactive oxygen species (ROS) generation**

For determination of ROS levels via fluorescent imaging, 4T1 cells were incubated for 2 h with 6 different groups under hypoxia or normoxia condition: (1) PBS, (2) NIR (0.5 W/cm^2^, 5min) +US (1 W/cm^2^, 3 MHz, 40 s) (3) Ce6+US (4) Ce6+PB@Hy (5) PB@Hy+NIR (6) Ce6+PB@Hy+NIR+US. The Ce6 concentration was 5 μg/mL in group 3, 4 and 6. Then, the fluorescent dye, DCFH-DA (10 μmol/L), was added and co-incubated for 20 min at 37 °C. Then, cells in group 2, 3, 5 and 6 were irradiated with the NIR or US. ROS level was determined by a confocal laser scanning microscope (CLSM; IX81, Olympus, Japan). The fluorescent intensity of each group was calculated by ImageJ software.

***In vitro* anti-tumor ability of PB+Ce6@Hy**

4T1 cells were seeded in 96-well plates at a density of 5 × 10^3^ cells per well and incubated for 24 h. Afterwards, 4T1 cells were incubated for 6 different groups under hypoxia or normoxia condition: (1) PBS, (2) NIR (0.5 W/cm^2^, 5min) +US (1 W/cm^2^, 3 MHz, 40 s) (3) Ce6+US (4) Ce6+PB@Hy (5) PB@Hy+NIR (6) Ce6+PB@Hy+NIR+US. The Ce6 concentration was 5 μg/mL in group 3, 4 and 6. Then, Then, cells in group 2, 3, 5 and 6 were irradiated with the NIR or US. At the end of the incubation, 5 mg/mL MTT PBS solution was added, and the plate was incubated for another 4 h. Finally, the absorbance values of the cells were determined by using a microplate reader (Emax Precision, USA) at 570 nm. The background absorbance of the well plate was measured and subtracted. The cytotoxicity was calculated by dividing the optical density (OD) values of treated groups (T) by the OD values of the control (C) (T/C × 100%). For live and dead assay, 4T1 cells were incubated with (1) PBS, (2) NIR (0.5 W/cm^2^, 5min) +US (1 W/cm^2^, 3 MHz, 40 s) (3) Ce6+US (4) Ce6+PB@Hy (5) PB@Hy+NIR (6) Ce6+PB@Hy+NIR+US. The Ce6 concentration was 5 μg/mL in group 3, 4 and 6. Then, cells in group 2, 3, 5 and 6 were irradiated with the NIR or US. Next, the cells were stained with flurescein diacetate (live, green) and propidium iodide (PI, dead, red) dye, and washed with serum-free Dulbecco’s modified eagle medium (DMEM). Finally, the images were captured by a confocal laser scanning microscope (CLSM; IX81, Olympus, Japan).

**Animal tumor models**

Female BALB/c mice aged 4-5 week were purchased from Vital River Company (Beijing, China). 100 μL of 4T1 cell suspension (1×10^6^ cells per mL) were subcutaneous injected into each mouse to establish the tumor models. The animal experiments were carried out according to the protocol approved by the Ministry of Health in People’s Republic of PR China and were approved by the Administrative Committee on Animal Research of the Wuhan University.

***In vivo* infrared thermography**

To monitor the *in vivo* photothermal effect, PB+Ce6@Hy (PB: 10 mg/kg, Ce6: 0.5 mg/kg) was intratumorally injected into the tumor-bearing mice, and then the tumors suffered from 0.5 W/cm^2^ irradiation for 3min at 0.5 h post-injection. PBS injection used as control group. Meanwhile, the temperature at the tumor was monitored using an infrared camera (Fotric 225).

***In vivo* hypoxia evaluation**

After the tumor size reached 200 mm^3^, the mice were divided randomly into 5 groups (n =3 per group): (1) PBS, (2) NIR (0.5 W/cm^2^, 5min) +US (1 W/cm^2^, 3 MHz, 40 s) (3) Ce6+PB@Hy (4) PB@Hy+NIR (5) Ce6+PB@Hy+NIR+US. Among them, the dose of PB in groups 3, 4 and 5 are 10 mg/kg. The dose of Ce6 in groups 3 and 5 are 1 mg/kg. The injection method is intratumoral injection. After 0.5h injection, the tumor site of mice in groups 2, 4 and 5 were treated by US or NIR. A total of 60 min later, the mice were all sacrificed, and the tumors should be effectively stained with HIF-1α.

***In vivo* antitumor study**

After the tumor size reached 200 mm^3^, the mice were divided randomly into 5 groups (n =3 per group): (1) PBS, (2) NIR (0.5 W/cm^2^, 5min) +US (1 W/cm^2^, 3 MHz, 40 s) (3) Ce6+PB@Hy (4) PB@Hy+NIR (5) Ce6+PB@Hy+NIR+US. Among them, the dose of PB in groups 3, 4 and 5 are 10 mg/kg. The dose of Ce6 in groups 3 and 5 are 1 mg/kg. The injection method is intratumoral injection. After 0.5 h injection, the tumor site of mice in groups 2, 4 and 5 were treated by US or NIR. Mice body weight and tumor volume in all groups were monitored every 5 days. A caliper was employed to measure the tumor length and tumor width and the tumor volume was calculated according to following formula. Tumor volume = tumor length × tumor width^2^ / 2. After 16 days treatment, mice were sacrificed. Five main organs (heart, liver, spleen, lung and kidney) of all mice were harvested, washed with PBS, and fixed with paraformaldehyde for histology analysis. The blood samples from these mice (≈1 mL) were collected for blood biochemistry analysis. And the tumor tissues were weighed, and fixed in 4% neutral buffered formalin, processed routinely into paraffin, and sectioned at 4 μm. Then the sections were stained with TUNEL and ROS.

**Statistical analysis**

Data analyses were conducted using the GraphPad Prism 5.0 software. Significance between every two groups was calculated by the Student’s t-test. *P < 0.05, **P < 0.01, ***P < 0.005.

Supplementary figures


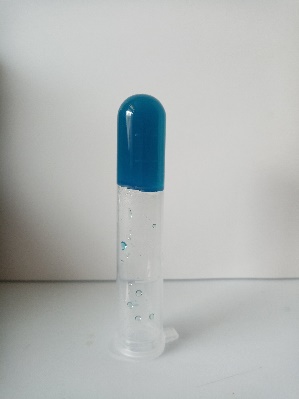


Fig. S1. Image of PB+Ce6@Hy.


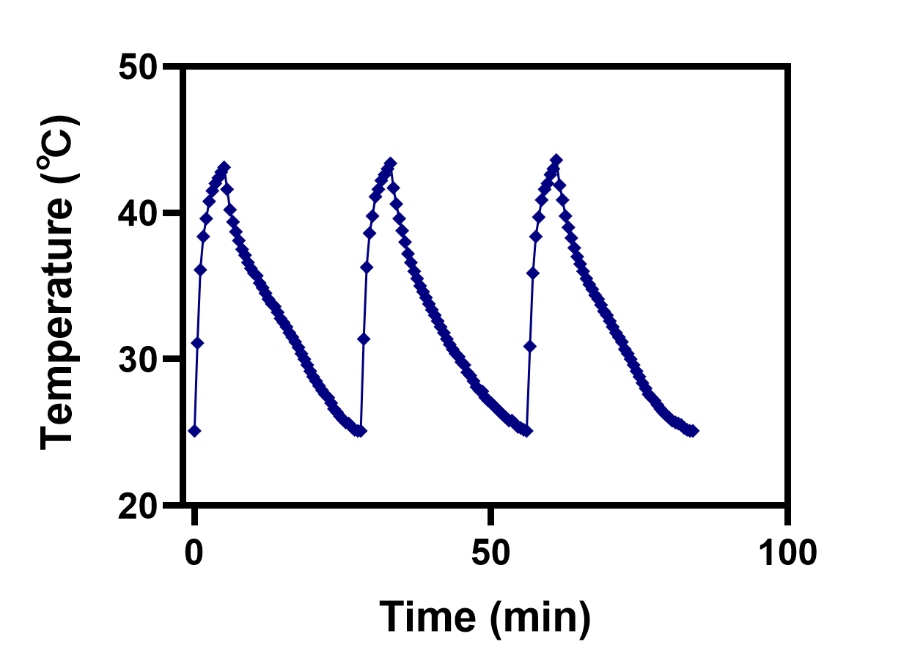


Fig. S2. Temperature variation of the PB nanoparticles solution at 100 μg/mL upon 3 repeated 808 nm NIR laser irradiation.


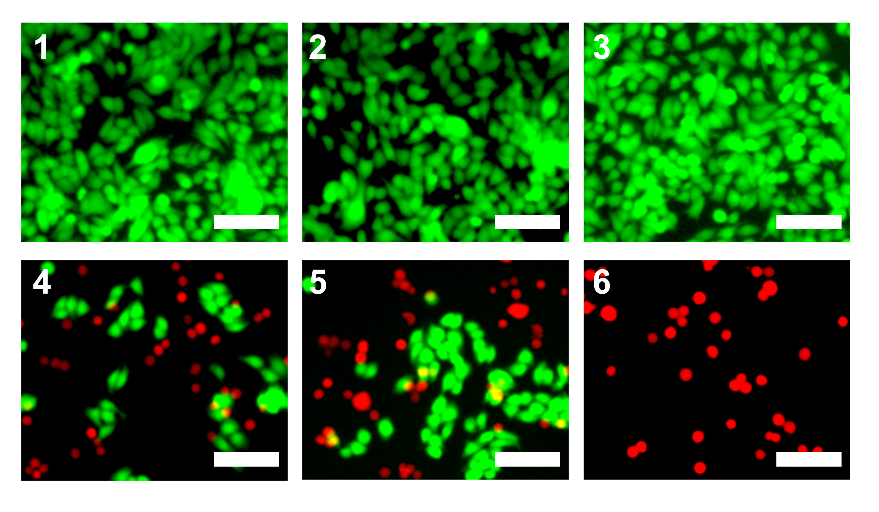


Fig S3. Fluorescence images of FDA/PI (scale bar = 50 μm) in 4T1 cells under different conditions.


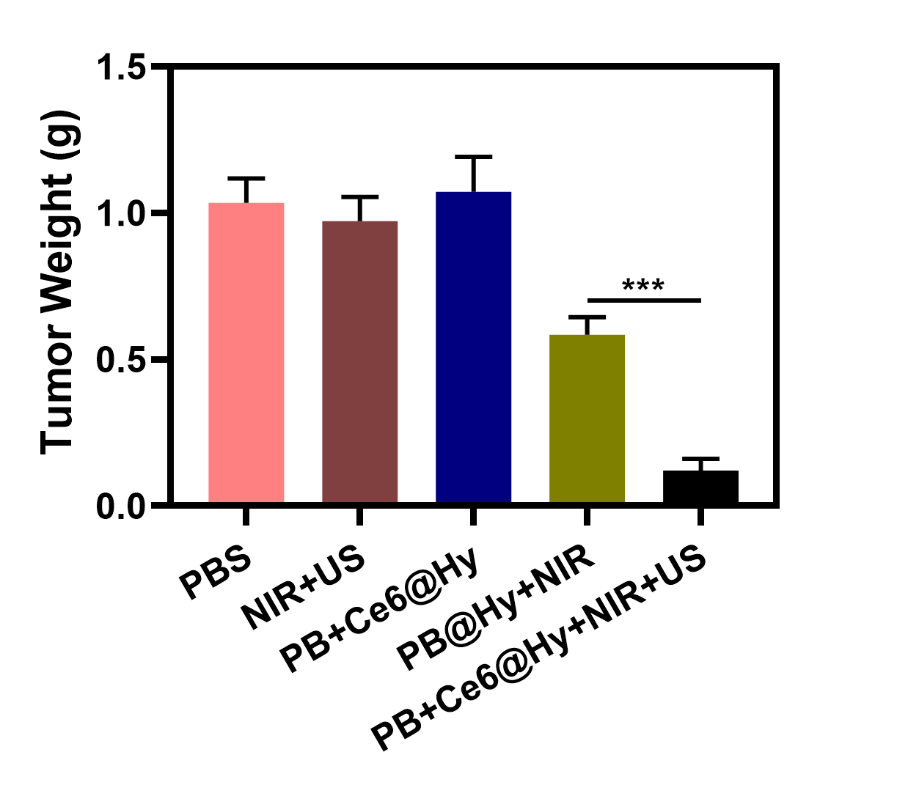


Fig S4. Evolution of the tumor weight during various treatments.

**References**

[1] D. Zhu, Z. Zheng, G. Luo, M. Suo, X. Li, Y. Duo, B.Z. Tang, Single injection and multiple treatments: An injectable nanozyme hydrogel as AIEgen reservoir and release controller for efficient tumor therapy, Nano Today 37 (2021) 101091.
